# Dyadic Intervention for Sexually Transmitted Infection Prevention in Urban Adolescents and Young Adults (The SEXPERIENCE Study): Protocol for a Randomized Controlled Trial

**DOI:** 10.2196/29389

**Published:** 2022-05-25

**Authors:** Maria Trent, Hasiya Eihuri Yusuf, Julia Rowell, Jacquelin Toppins, Colin Woods, Steven Huettner, Camille Robinson, Errol L Fields, Arik V Marcell, Ralph DiClemente, Pamela Matson

**Affiliations:** 1 Department of Pediatrics Division of Adolescent/Young Adult Medicine Johns Hopkins University School of Medicine Baltimore, MD United States; 2 Department of Social and Behavioral Sciences New York University School of Global Public Health New York, NY United States

**Keywords:** STI, adolescent, young adult, youth, dyadic, sexual risk, heterosexual, health education, sexually transmitted disease, disease, adolescent, education

## Abstract

**Background:**

Adolescents and young adults (AYA) aged younger than 25 years have the highest rates of sexually transmitted infections (STIs) in the United States. Current STI prevention strategies for AYA rely primarily on individual approaches, leaving sexual partners with significant unmet sexual and reproductive health care and health education needs. Dyadic interventions may hold promise for harnessing the power of communal coping within relationship dynamics to enhance sexual decision making, communication, and behavior changes that reduce the future risk of STIs.

**Objective:**

This paper describes the protocol and research methods of a dyad-based behavioral intervention that augments individual evidence-based interventions with joint health education counseling for heterosexual AYA dyads within a primary care setting. The trial aims to improve partner communication and collaborative sexual decision making and promote the adoption of sexual behaviors such as consistent condom use. The primary objective of this study is to assess the feasibility, acceptability, and effectiveness of a dyadic intervention targeted at preventing STIs in heterosexual couples in an urban setting.

**Methods:**

A total of 100 AYA (50 dyads) aged 16 to 25 years, engaged in heterosexual intercourse, who reside in the city and are willing to recruit their main sexual partner for the study will be recruited and randomized into 2 groups, an intervention arm and a control arm. Participants will be recruited from an AYA medicine clinic and by using social media (Facebook and Instagram). The index participant and partner will complete a single individual session separately (Sister to Sister or Focus on the Future) with a gender-matched health educator. Dyads will then be randomized to receive an additional joint debriefing session together to discuss relationship dynamics, condom negotiation, etc. Participants will separately complete a telephone interview 6 weeks postintervention to determine the feasibility, acceptability, and impact of the intervention on mutual sexual negotiation, consistency of condom use, and communal coping skills, etc.

**Results:**

So far, 25.4% (44/173) of eligible participants have been enrolled and randomized. Participants are mostly female (20/22, 91%), with at least a high school diploma (19/22, 86%), and 9 average lifetime sexual partners. Acceptability is high, with 98% (43/44) of participants expressing satisfaction with their study experience; 100% of dyads recruited were still together at 6-week follow-up.

**Conclusions:**

Findings from this study will add to the current literature on the approaches to STI prevention, and its success will inform its application in risk reduction counseling for youth who are most at risk.

**Trial Registration:**

Clinical Trials.gov NCT03275168; https://www.clinicaltrials.gov/ct2/history/NCT03275168

**International Registered Report Identifier (IRRID):**

DERR1-10.2196/29389

## Introduction

Sexually transmitted infections (STI) have been on the rise in the United States, growing to epidemic proportions among adolescents and young adults (AYA) aged 15 to 24 years [1]. As a group, AYA account for the highest rates of STI, contributing more than 50% to the $16 billion in lifetime cost of the infection [2,3]. In addition to the heightened risk of STIs, AYA residing in low-income, densely populated urban communities experience racial and ethnic disparities and often have limited access to STI screening, treatment, and other sexual and reproductive health needs [4,5]. AYA have also been characterized as a vulnerable population due to cognitive factors such as inaccurate risk perceptions, increased biologic susceptibility (eg, cervical ectopy), engagement in unprotected intercourse, and sexual partnership patterns such as serial monogamy and concurrency [6-9]. National surveillance of the high school youth in the United States show that more than half of high school students are sexually active [9], and without access to services, they may suffer STI-associated sequelae. Despite the emerging public health threats and incidence of STIs in this age group, many AYA are not tested for STIs [10]. While the Centers for Disease Control and Prevention (CDC) guidance on the approach to STI prevention in youth is clear about the need for developmentally appropriate approaches to care, youth continue to face barriers to accessing sexual and reproductive health clinical services and education.

STI prevention efforts have primarily relied on individual approaches and heavily target girls and young women in heterosexual relationships, leaving young men with significant unmet sexual and reproductive health needs [11-13]. Young women are also diagnosed and treated for STIs more often compared to young men, who are often asymptomatic. As such, partner notification and treatment is a key strategy for disease control. Individuals with bacterial STIs often notify and refer their partners for treatment, but the relationship context influences the timing, manner of communication, and outcome of notification. Even so, less than half of male partners in a heterosexual relationship notified for STI treatment are successfully treated [12]. In response to this public health failure, many states are establishing regulations to implement expedited partner therapy [14-16]. In addition, the US Preventive Task Force recently recommended the integration of behavioral counseling as part of STI management, especially for adolescents at higher risk for new or recurrent disease [17]. This clinical change is estimated to have a moderate benefit and will reduce the probability of acquiring new STIs. The context for STI treatment services has also shifted from public health departments to primary care settings. Unfortunately, national data suggest that less than half of eligible patients are screened for STIs and many primary care providers serving youth fail to provide needed services [10,11,18]. Given the personal and societal costs associated with STIs, new models of care that integrate effective public health interventions into primary care settings are warranted. Public health departments have effectively used individual health educator models for STI/HIV risk reduction counseling but with limited effectiveness due to limited partner engagement. Efforts for engaging sexual partners of AYA using an integrated approach that combines behavioral interventions and health education and other methods are needed as individual level approaches fail to harness the power of joint intervention to improve sexual decision making and behavior.

Sexual relationships among AYA have long been believed to be delicate, short-lived, and lacking in commitment and mutual trust as compared to adult sexual partnerships. Evidence to the contrary proves that although youth, particularly males, tend to engage in extra-dyadic sex (sex with someone other than their partners) [6] and have higher tendencies toward concurrency and serial monogamy [19], STI in AYA is more nuanced and complex, often occurring in the context of committed relationships with or without concurrency. AYA relationships possess levels of trust and engagement, qualities that are relevant for and amenable to modifications for improving communication and promoting emotional and sexual satisfaction and negotiation skills. Such interventions can culminate in better self-management and a reduced risk of acquiring or transmitting STIs. Among adults, engagement of couples (or sexual dyads) for STI/HIV prevention have proven effective [20,21]. Research with AYA suggests that understanding early relationships may help prevent STIs but that we have failed to use our knowledge about the structure and function of romantic relationships occurring during this critical developmental learning period to engage youth for individual and dyadic behavior change. Formation of romantic relationships is a key task of adolescence with significant implications for young adult adjustment and health outcomes [22-24]. Male partners can be engaged through partnerships with female sexual partners [13]. Adolescent sexual dyads are formative and sufficiently stable for dyadic intervention, with relationships lasting 8 months on average [22]. Recent research with adolescent dyads also demonstrates that male and female partners report shared decision making and strong feelings toward their partners [24,25]. A dyadic approach to STI prevention allows both parties to see mutual responsibility for protecting each other from STIs, facilitates them working together, highlights the context and connection to disease acquisition, and provides a safe space for communication about difficult issues such as concurrency, while giving room for the dyad to learn key communication skills and receive support and guidance from a health professional. Although several studies demonstrate the effectiveness of adult-focused couple interventions for STI/HIV prevention, no published studies address the AYA dyad as the unit of transformational change or have contextualized the interpersonal dynamics influencing individual sexual health behavior change within young romantic partnerships. To address this issue, the Sexperience study was developed using the tenets of transformational motivation theory to bolster the integrative model of behavior change as a conceptual framework [26,27]. The aim of this study is to examine the feasibility, acceptability, and effectiveness of a partner intervention in a community facing significant STI/HIV health disparities.

## Methods

### Study Design

**Figure 1:**
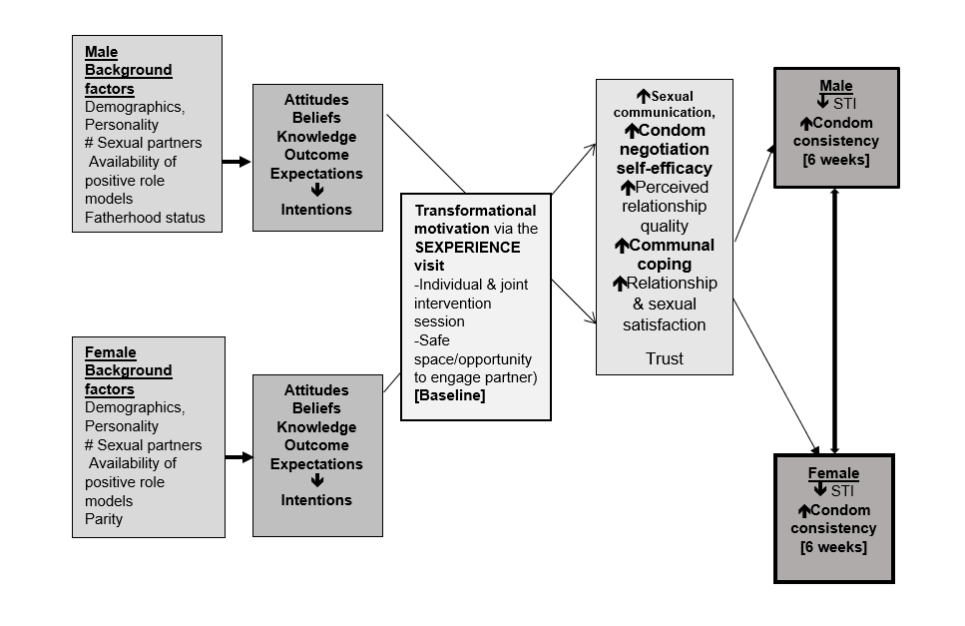
Conceptual framework of the Sexperience intervention.

The Sexperience study is an exploratory randomized controlled trial designed to examine the feasibility, acceptability, and differences in short-term behavioral outcomes between youth who receive both individualized counseling and joint debrief with sexual partner and those who receive sexual risk reduction counseling alone. The study question operationalizes the tenets of transformational motivation theory [27] as a part of the conceptual framework (Figure 1) based on the integrative model of behavior which asserts that being in a relationship can change the member’s behavior from one of self-centeredness to one that is driven to improve the relationship and is health enhancing. The integrative model of behavior serves as a backdrop for integrating the transformational motivation theory for dyadic research with AYA as a strategy to reach both female and male patients for treatment and preventive services [26]. The experience gained and lessons learned from previous research are invaluable tools for achieving the desired goals of this study [28].

**Figure 2:**
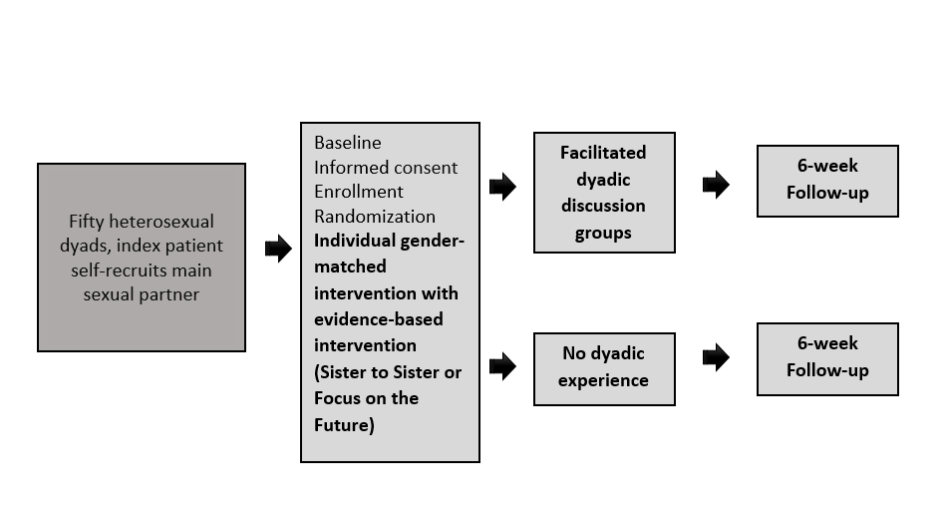
Overview of Sexperience study design.

A total of 50 AYA dyads aged 16 to 25 years, engaged in heterosexual intercourse, who reside in the greater Baltimore (Maryland) metropolitan area (BMA) and are willing to recruit their main sexual partner for the study will be recruited and randomized. Participants are being recruited from an academic adolescent/young adult medicine practice and the BMA using social media (Facebook and Instagram). The index participant and their recruited partner complete an individual behavioral intervention session separately (Sister to Sister or Focus on the Future) with a gender-matched health educator. Dyads are then randomized to completion or to receive an additional dyadic debriefing session. Participants separately complete an exit survey at the end of the intervention and a telephone interview 6 weeks postintervention to determine the feasibility, acceptability and perceived impact of the intervention on mutual sexual and condom negotiation, consistency of condom use, and communal coping skills (Figure 2).

### Inclusion Criteria

Index participants are eligible if they are aged 16 to 25 years, live in the BMA, agree to recruit their main partner to the study, engage in male-female (heterosexual) intercourse, are willing to participate in a single individualized session with a health educator followed by a joint intervention, are willing to be contacted in 6 weeks for a telephone interview, have no reports of intimate partner violence with their current sexual partner, and have a current sexual partner within 5 years of their age. The partner must be aged 16 to 30 years based on adhering to prespecified age differentials to coincide with state law and developmental expectations, engage in male-female (heterosexual) intercourse, permanently reside in the BMA, and be willing to participate in a single individual session with health educator with or without a dyadic debriefing session with their partner and both health educators depending on randomization.

### Exclusion Criteria

Index participants and partners who are unable to communicate with staff or participate in study procedures due to cognitive, mental, or language difficulties will not be eligible for recruitment into the study. Dyads will also be excluded if in same-sex main partnership or a member of the dyad is currently enrolled in another sexual behavior study, the patient/partner is currently pregnant, one or both partners has a known concurrent HIV infection, one or more partners has a pending incarceration, if there is more than 5 years age difference between the two partners, or there is evidence of intimate partner violence in the relationship.

### Setting

Baltimore, Maryland, is the primary site of this study. Baltimore is a large city whose citizens face significant STI and HIV disparities. Baltimore was ranked the city with the highest STI rates by the CDC in 2020 [29]. The Maryland Department of Health and Mental Hygiene has determined that every county is a chlamydia hot spot, and county maps demonstrate that among hot spots, the city of Baltimore is the most densely affected. Although there are extremely low rates of youth noninsurance, the use of primary care providers for routine health maintenance drops off considerably. The Adolescent and Young Adult Clinic at Johns Hopkins established a Title X reproductive health clinic to serve the youth of Baltimore to aid in resolving the reproductive health access issues facing urban youth. The clinic sees over 200 unduplicated positive STI cases and partner referrals annually. Patients may also be referred for treatment through the Johns Hopkins Wellness Center, the Johns Hopkins Pediatrics Emergency Department, or the Baltimore City Health Department clinical sites.

### Recruitment, Informed Consent, and Randomization

Recruiters will be available via phone during office and some evening hours in the clinic. Patients will be referred for study contact by their providers. Patients who agree to be contacted will be approached by a recruiter in person or via phone and if eligible, the dyad will be scheduled for written informed consent and intervention procedures. After enrollment, participants will be assigned to the control group or intervention group using a computer-generated randomization assignment. Envelopes based on dyadic study group number will be available to the research staff at the time of enrollment to adhere to the randomization sequence.

### Sexperience Intervention

Following contact of prospective participants via social media or through the AYA practice by a member of the research team or the research coordinator, willing participants who agree to invite their sexual partners will be enrolled into the study when they bring their partners along to the clinic on a specified date (usually within 2 weeks of agreeing to be in the study). Researchers will provide information to participants regarding the study and its processes individually and then seek informed consent. All participants who sign the informed consent with their partners will be randomized to either the intervention arm or control arm. Integral to the Sexperience intervention is the use of effective behavioral interventions designed to reduce STI/HIV risk behaviors, Focus on the Future and Sister-to-Sister, which will be used for male and female participants, respectively.

The Focus on the Future intervention [30] is an individual-level single session intervention designed to reduce the spread of heterosexually transmitted STI and HIV in African American males. This is achieved through education on correct and consistent condom use and motivation for sexual behavioral change. Sister to Sister [31] is a similar intervention designed for heterosexual females and aims to reduce the risk of HIV and other STI acquisition in AYA women. Sister to Sister, like Focus on the Future, is a 1-time brief intervention that encompasses training for skills building in sexual negotiation, demonstrations on the right way to insert a condom, and role playing. Both of these interventions have been shown to be effective in reducing STI risk and improving healthy sexual behavior.

The novel component of this intervention is that sexual partners will come together at the end of the individual sessions for a joint debriefing session that includes viewing of the condom negotiation videos and role-playing communication for condom use. At the end of the session with the Sexperience health educators, the patient will be able to state/demonstrate (1) understanding of the definition of STIs, (2) how to prevent future STI episodes, (3) proper use of male and female condoms, and (4) use of effective communication and condom negotiation strategies with their partner. Low-resource patients have been receptive to health educator support, and our preliminary studies indicate that improved communication between an adolescent and her partner occurs with the support of a professional who can educate and motivate adolescents with an STI and provide expertise on STI management. Video/audio recording of dyadic intervention sessions will be used to ensure intervention delivery is consistent with the protocol and to identify content themes to bolster our understanding of relationship dynamics and the potential of communal coping.

Text message boosters related to care-seeking behavior, condom use, and condom access will be provided over the 6 weeks of the study. Messages will be standardized and delivered via an automated 1-way system. No protected health information is disclosed as a part of this communication, but youth will be encouraged to provide an extra layer of protection on existing cell phone security to allow for an additional degree of privacy related to message content. Welcome messages will be sent right after enrollment, “Welcome to the Sexperience study,” followed by 1 text every week, including: “Don’t forget to try out your new communication skills,” “Did you practice the Sexperience role play? Text the # of times you practiced together,” “Condoms prevent STDs. Stop by the clinic if you need some,” “Condoms can improve pleasure, if you need some stop by the clinic,” and “Want to up your game? Practice your condom techniques.” In week 6, we will send “Thanks for trying Sexperience, we’ll call you soon for feedback.”

SMS messages will be sent through TextNow for communicating with participants in both arms of the study. TextNow is one of the fastest growing technology companies with strict policies to protect the user by preventing unauthorized disclosure of information and ensuring physical, electronic, and procedural safeguards are in place according to industry standard procedures and security procedures. Finally, all participants will be encouraged to use standard safety mechanisms such as a pin or password to lock access to their cell phones by others.

Participant dyads in the control group will also receive the 20-minute Focus on the Future for male and Sister-to-Sister for female participants and one-on-one sessions with a gender-matched health educator to guide the patient through skills-based risk reduction counseling. As noted above, these interventions have been evaluated (with African American males and females residing in communities with high STI rates) and were found to increase condom use, reduce unprotected intercourse, and reduce new STIs among participants and are considered effective interventions for clinical implementation by the CDC. Participants in the control arm will also receive positive health-related attention-control text messages focused on general health (healthy eating, exercise, spending time with family and friends, and healthy sleep). No text messages related to relationships or condom use will be sent. As with the intervention group, the message “Thanks for trying Sexperience, we’ll call you soon for feedback” will be sent in 6 weeks. None of these texts will request a response.

### COVID-19 Procedure Adjustments

Due to the pandemic, the remainder of participants will be recruited and enrolled virtually. Social media will be used for recruitment, and Zoom videotelephony software will be used to complete the enrollment visit. Recruiters will screen potential recruits over the phone and if eligible, set up a Zoom meeting for enrollment. Participants will be consented with the oral consent script. Recruiters will read the baseline Audio Computer-Assisted Self-Interviewing to the participants and record their answers. There will be separate Zoom meetings for the individual gender-matched intervention, and if the participants are randomized to the intervention group, they will join after for the joint debrief session. The exit survey through Qualtrics will be read to the participants afterward by the recruiters, and their answers will be recorded. Gift cards will be mailed to participants after the enrollment visit has been completed. The 6- to 8-week follow-up survey will remain the same and be completed over the phone with participants.

### Outcomes

The primary outcome variable for this pilot study is feasibility of recruitment. Recruitment feasibility will be defined as the number of dyads completing the intervention out of the number of eligible dyads. The feasibility of short-term retention will be calculated as the proportion of enrolled participants who complete a follow-up interview at 6 weeks.

The secondary outcome variable for this pilot study is acceptability, measured as participant satisfaction with their intervention experience. Participant satisfaction will be measured using a brief survey asking participants to rate their experience with qualitative feedback documented.

### Sample Size Determination and Statistical Analysis

A total of 50 adolescent dyads will be enrolled. The choice of 50 dyads is inherently arbitrary and was the number considered realistically feasible to enroll within the limited time of this pilot study. While the study will lack statistical power to detect significant differences in outcomes between the two study arms, point estimates derived from this work will be invaluable for future research proposals to evaluate efficacy. Feasibility and acceptability will be evaluated against benchmarks from previous studies. Observed recruitment rates from the proposed pilot study will be compared to expected recruitment rates (determined from previous studies that recruited adolescents with an STI diagnosis). Prior studies recruiting from the AYA clinic have achieved recruitment rates ranging from 48% to 91%. A similar approach will be used to compare satisfaction scores to an a priori benchmark of patient satisfaction. Based on previous work, average patient satisfaction greater than 80% would be necessary to conclude that the intervention is acceptable. Descriptive statistics will be used to make a comparison of the baseline characteristics between the intervention and control group. Statistical analysis will be based on the standard for randomized controlled trials, and both primary and secondary outcomes for each group will be assessed using an intention-to-treat analysis. Data distribution will be assessed, and comparisons of the 2 groups will be conducted using generalized estimating equations. *P*<.50 will be considered statistically significant. The multiple imputations method will be used to address missing data.

### Risks and Ethical Considerations

There are no significant medical risks involved in this study. While the study will attempt to preserve patient confidentiality, there is potential for breaches of confidentiality, but it is miniscule given the limited number of personnel with access to participant information. Finally, while the Sexperience intervention focuses on positive communication and condom negotiation, dyads may experience conflict or disagreement during the context of the joint session. While the health educator will be trained in basic conflict resolution, there are staff onsite to provide both mental health (social work, mental health counselors, psychologists) and seasoned security staff available to intervene as needed. There are no additional risks associated with these telephone calls.

Human subject approval has been granted for beta testing of this research protocol by the Johns Hopkins institutional review board (IRB00083609), and the study was registered at ClinicalTrials.gov [NCT03275168]. All patient data are kept confidential and secure. Individual patient data shared during health education sessions and clinical visits (sexual history data, lab results, clinical findings) will not be disclosed to the partner in accordance with Health Insurance Portability and Accountability Act regulations. However, the patient will be encouraged to notify partners regarding behavioral risks that impact their partner per standard of care and public health practice. The research team on this project has training and experience in the protection of human research participants and the treatment of protected health information. All staff have completed human subjects training through the institution’s research compliance training program as a part of orientation training and as a condition of their employment.

### Data Safety and Monitoring

The study will use a data safety monitoring plan consistent with National Institutes of Health guidelines. The research team will meet regularly to communicate about ethical issues related to the study. A data safety and monitoring committee was created; members have no formal association with the study and their selection is based on their expertise in AYA clinical interventions, sexual and reproductive health service delivery, and biostatistics.

### Payment, Remuneration, and Benefits

Study incentives are US $50 for the enrollment visit and US $50 for the 6-week follow-up interview for each participant, both patient and partner. Those participants who elect to complete the in-depth interview will receive an additional US $50. There are no anticipated costs to participants that are unaccounted for in the support and remuneration plan for the study or usual care. Transportation support may be provided for participants with transportation issues. There may be no direct benefits to participants in this study.

## Results

**Figure 3:**
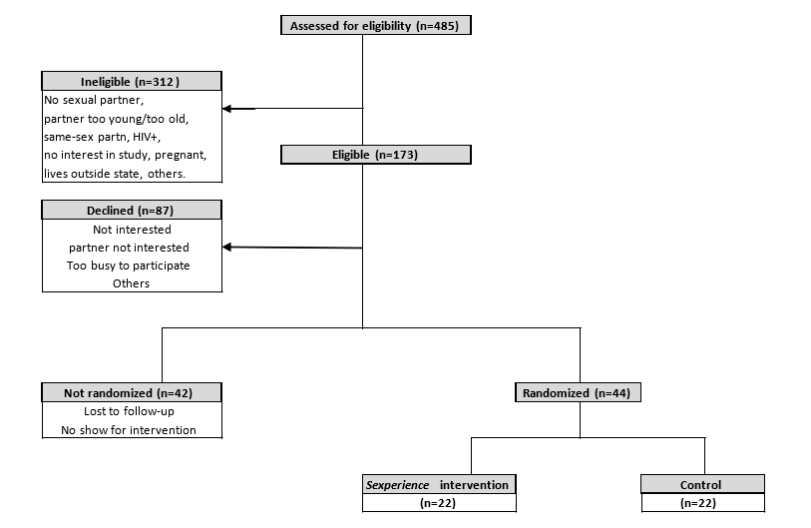
Recruitment of study participants.

Study recruitment began in January 2018 and was initially estimated to be completed in February 2021. As of March 30, 2021, 485 persons were assessed for eligibility, of which 312 (64.3%) were ineligible. Of the 173 eligible participants, 87 (50.3%) declined to participate and 42 (24.3%) were not randomized due to certain factors (Figure 3). So far, 22 dyads (n=44) have been recruited. Of the index patients randomized, most were females (20/22, 91%), with at least a high school diploma (19/22, 86%), and an average of 9 lifetime sexual partners. Index patients and their partners did not differ in age, with mean ages of 21.7 (SD 2.6) years and 22.6 (SD 2.5) years, respectively, and age at first sexual encounter 15.4 (SD 2.5) years and 15.1 (SD 1.8) years, respectively.

## Discussion

### Summary

Preliminary findings from the study show that a dyadic intervention for heterosexual AYA is both feasible and acceptable. To our knowledge, this study will be the first of its kind to engage AYA dyads in a joint STI risk counseling intervention. While many current research studies have used couples engagement for prevention of sexually transmitted diseases, most have focused primarily on HIV to the neglect of other STIs (*Neisseria gonorrhoeae*, *Chlamydia trachomatis*, *Trichomonas vaginalis*), which account for the preponderance of STIs [20,32,33]. Similar studies have been limited to adults or male-male partnerships to the exclusion of younger age groups and heterosexual dyads [9,12,13]. The results from this critical inquiry will further clarify the potential role for a joint health educator–administered sexual education and counseling intervention in enhancing AYA access to sexual and reproductive health services in primary care settings and will determine the feasibility, acceptability, and effectiveness of using the sexually involved dyad as a vehicle for behavioral change. It will also give us an insight into the role of actively engaging sexual partners in joint communications for promoting healthier sexual behaviors on STI prevention compared to the general practice of individualized risk reduction counseling, partner notification, and expedited therapy without mutual engagement [14,17,34]. Dyadic interventions have been used in many medical conditions including parent-adolescent dyads for improving HIV prevention and self-efficacy [35], obesity prevention, and smoking and sedentary lifestyle cessation and were influential for sparking behavior changes [36] and preventing HIV transmission between serodiscordant same-sex male couples. A dyadic intervention for heterosexual couples in Uganda was equally effective for HIV prevention [32].

Consistent condom use and condom negotiation skills reduce STI transmission from an infected partner. Given the high impact of STIs among AYA across the United States, this study will be a first step toward the use of previously untested strategies and a remarkable addition to the current armamentarium in the fight against high STI rates in minority groups and associated sequelae. The study findings will provide new information on more effective ways to engage sexually active AYA in a discussion on sexuality and skills building for a healthier and safer life.

### Limitations

The study procedures target African American youth living in an urban community that is currently ranked number 1 in STI rates by the CDC. While we acknowledge that this population may not reflect the distribution of AYA across the United States, the distribution of STI is mirrored in the demographics of study participants. This initial study, although not generalizable to the general US population, is a first step toward larger and more heterogeneous studies.

Since its inception, the study has been modified to accommodate challenges that were not anticipated prior to the launch of the study. One such challenge was the problem with the clause that restricts study recruitment to only persons with a previous or active STI diagnosis. This was met with difficulty in recruiting persons who were otherwise eligible for the study but expressed reluctance in volunteering STI information. Based on the this, a decision was reached to remove previous (or current) STI as a prerequisite for study enrollment. This modification to the study design was approved by the institutional review board and is consistent with our approach to this exploratory study, and its effect on the speed and ease of recruitment will be evaluated at the end of the study.

Additionally, the current COVID-19 pandemic has been a major hindrance to the recruitment of participants into the study, as recruitment has been conducted through the clinic and via social media advertisements and interventions were previously delivered at in-person clinic visits. In response, the remainder of participants will be recruited and enrolled virtually. Social media will be used for recruitment and Zoom will be used to complete the enrollment visit. Study visits have also been moved to virtual meetings. There will be separate Zoom meetings or rooms for the individual gender-matched intervention and, if the participants are randomized to the intervention group, they will join their partners in the main Zoom room for the joint debrief session. Both participants recruited prior to the onset of the pandemic and during the pandemic will be followed up using phone interviews as designed. The effect of the COVID-19 pandemic on study recruitment or outcomes will be evaluated at the end of the study.

### Conclusion

In conclusion, engaging heterosexual urban AYA dyads in a joint intervention for STI prevention and improving self-efficacy for sexual risk reduction is both feasible and acceptable. If successful, the outcome of this study will add to the current literature on the approaches to STI prevention and its success will inform its application in risk reduction counseling for youth who are most at risk for STI in the United States.

## Abbreviations

AYA: adolescents and young adults
BMA: Baltimore (Maryland) metropolitan area
CDC: Centers for Disease Control and Prevention
STI: sexually transmitted infections
